# How the Invisible Hand is Supposed to Adjust the Natural Thermostat: A Guide for the Perplexed

**DOI:** 10.1007/s11948-016-9780-3

**Published:** 2016-04-26

**Authors:** Servaas Storm

**Affiliations:** Department of Economics, Faculty TPM, Delft University of Technology, Jaffalaan 5, 2628 BX Delft, The Netherlands

**Keywords:** Climate change economics, Carbon pricing, Social discount rate, Equity versus efficiency, Normative uncertainty

## Abstract

Mainstream climate economics takes global warming seriously, but perplexingly concludes that the optimal economic policy is to almost do nothing about it. This conclusion can be traced to just a few “normative” assumptions, over which there exists fundamental disagreement amongst economists. This paper explores two axes of this disagreement. The first axis (“market vs. regulation”) measures faith in the invisible hand to adjust the natural thermostat. The second axis expresses differences in views on the efficiency and equity implications of climate action. The two axes combined lead to a classification of conflicting approaches in climate economics. The variety of approaches does not imply a post-modern “anything goes”, as the contradictions between climate and capitalism cannot be wished away.

## Introduction

The contrast is striking. While climate science is sending out loud-and-clear messages that fossil-fuel disinvestment must start now, letting go of coal and oil and diverting resources into renewable energy technology systems, to keep warming below the 2 °C limit (IPCC 2014), mainstream climate economics claims that overly ambitious climate targets will unnecessarily hurt the economy and immediate de-carbonization is too expensive. Most climate economists thus recommend humanity to just wait-and-see. Their idea is that it makes sense to set the initial carbon price at a low level and then ratchet it upward (Nordhaus 2007, 2008, 2010; Tol 2013; Golosov et al. 2014; Gerlagh and Liski 2013). It is “efficient” to now allocate supposedly scarce resources to other, higher-yielding, investment purposes (than climate mitigation) and use the returns thus generated to finance more cost-effective climate mitigation technologies in the future. This approach rests on a social welfare function, derived from neoclassical economics, which discounts future economic goods using a social discount rate, consistent with current market rates of return (Nordhaus 2008, 2010; Tol 2009, 2014). Then later, when the carbon price is ramped up, producers and consumers are in a position to shift more easily to those alternatives, making the higher price economically less disruptive. Further support for this “climate policy ramp” is given by expectations that greenhouse gas (GHG) mitigation options will become cheaper in future, while climate-change losses are increasing more than proportionally with temperature change (Gerlagh and Liski 2013). The economics consensus on the slow-ramp is strong, as Nordhaus (2007, p. 687) writes: “The findings about the climate-policy ramp have survived the tests of multiple alternative modeling strategies, different climate goals, alternative specifications of the scientific modules, and more than a decade of revisions in integrated assessment models.” Tol (2013), in an article which has been taken by some as a definitive summary of what economics has to say about climate change, concludes that “a convincing alternative to the intuitively incorrect conclusion that continued warming is optimum, is still elusive.”^1^ This paper challenges this consensus on the “climate policy ramp” following Heal and Millner’s (2014) innovative work on “normative uncertainty”—by highlighting the inescapable value judgements, which underpin this consensus as well as alternative economic analyses of climate change, and the need for new normative decision frameworks But before doing so, the next section first locates the discussion of normative uncertainties within the context of conventional economic analysis of global warming and climate policy.

## Misplaced Concreteness and How to Avoid It

Economists and governments generally use dynamic integrated-assessment models (IAMs) to evaluate the long-run benefits and costs of policies to slow down global warming. IAMs for climate change are multi-equation, mostly deterministic but sometimes probabilistic, computerized models linking aggregate and long-run economic growth with simple climate dynamics in order to analyze the economic impacts of the GHG-driven externality of global warming. Prominent competing IAMs include MERGE (Manne et al. 1995), PAGE (Hope 2006a, b), FUND (Anthoff and Tol 2012), and DICE/RICE (Nordhaus 2008; Nordhaus and Sztorc 2013), which is probably the most widely used climate model. The relative merits and limitations of these models are widely discussed and debated, and a thriving cottage industry has grown up producing model variations, extensions, and sensitivity analyses. This literature on IAMs is beyond reviewing—but recent contributions^2^ hold that all IAMs have in-built unrealistic assumptions on economic growth, climate damage functions and climate risks which together result in a gross underassessment of the actual probability of unmanageable—catastrophic—climate change. The problems are endemic as well as intrinsic, as Pindyck (2013, p. 860) argues: “These models have crucial flaws that make them close to useless as tools for policy analysis: certain inputs (e.g., the discount rate) are arbitrary, but have huge effects on the social cost of carbon (SCC) estimates the models produce; the models’ descriptions of the impact of climate change are completely ad hoc, with no theoretical or empirical foundation; and the models can tell us nothing about the most important driver of the SCC, the possibility of a catastrophic climate outcome. IAM-based analyses of climate policy create a perception of knowledge and precision, but that perception is illusory and misleading.” IAMs in particular ignore the non-negligible probability of catastrophic warming, even if this low probability is not objectively knowable. IAMs focus on median temperature change, not on the risks of temperature extremes, but as recent data from Wagner and Weitzman (2015) show, even at relatively low increases in median temperatures (of say 3.4 °C), the risk of eventual temperatures exceeding 6 °C rises rapidly to more than 10 %.“… the artificial crispness conveyed by conventional IAM-based [cost-benefit analysis] is especially and unusually misleading,” writes Weitzman (2009, pp. 26–27), continuing that “we may be deluding ourselves and others with misplaced concreteness …” and concluding that “perhaps in the end the climate-change economist can help most by not presenting a cost-benefit estimate for what is inherently a fat-tailed situation with potentially unlimited downside exposure as if it is accurate and objective and perhaps not even presenting the analysis as if it is an approximation to something that is accurate and objective ….”

**Fig. 1:**
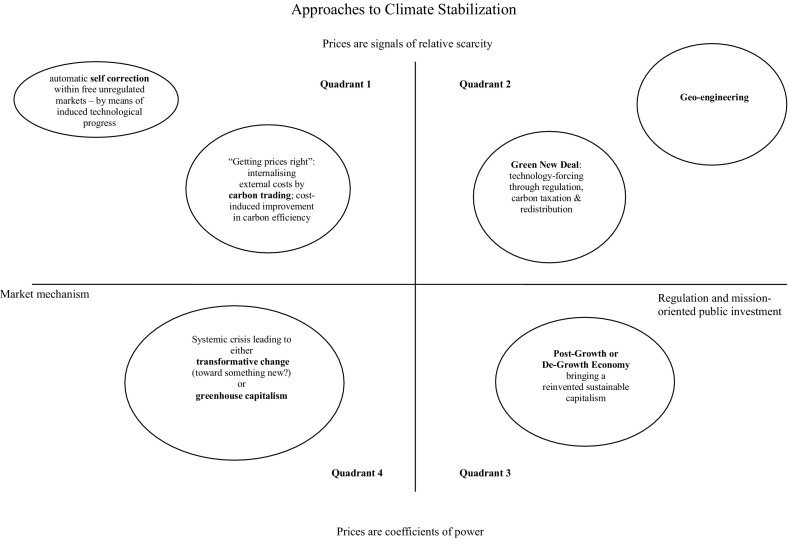
Approaches to climate stabilization

Climate science and economics are unable to deal with the multiple “unknown unknowns” of climate change—and have to come to terms with the fact that they cannot predict the extent and (physical and economic) consequences of global warming to any acceptable degree of accuracy. Improving IAMS, “is not helpful in the face of catastrophic risks and deeply uncertain probabilities of worst-case scenarios,” write Scrieciu et al. (2013, p. 157), “economies are highly complex non-linear systems and it is *impossible* [emphasis added] to accurately predict their future evolution.” It makes more sense to look more closely into the multiple uncertainties associated with climate change—which is exactly what Heal and Millner (2014) do, when they decompose irreducible uncertainty (associated with global warming) into “scientific” versus “socio-economic” uncertainty. Scientific uncertainty, in their definition, arises from our incomplete knowledge of the unfathomably complex climate system (e.g. Allen et al. 2006; Archer 2009)—the experts’ disagreement on the famed “climate sensitivity parameter” is a prominent case-in-point (Millner et al. 2013; Knutti and Sedlacek 2013), while model outcomes’ sensitivity to initial conditions is another example (Dietz and Fankhauser 2012). However, even if all scientific uncertainty were resolved (in a probabilistic manner), we would still face major “unknown unknowns” concerning (a) the impacts of climate change on human societies; (b) the responses of these societies to future climate impacts (one key issue here is whether low-carbon and/or carbon-free (energy) technologies will develop fast enough and can be scaled up quickly enough);^3^ and (c) the reactions of these same societies to future climate change mitigation policies. Heal and Millner (2014) treat a part of these socio-economic uncertainties as “positive” uncertainty, assuming that the disagreement in experts’ views arising from a general lack of empirical knowledge of how socio-economic systems work will be overcome once we know more. More actuarial studies based on actual climate damage could, for instance, help reducing the uncertainty associated with point (a) over time (Gorvett 2014). Likewise, empirical research may teach us more about how long it will take to develop and implement critical zero-carbon technologies.^4^ But part of the socio-economic uncertainty must be qualified as irreducible “normative uncertainty” which arises from differences in fundamental value judgements, not just over key welfare parameters such as the pure rate of time preference and the elasticity of the marginal utility of consumption (Arrow et al. 2012; Gollier 2012), but more importantly over the way(-s) the larger economic system works and what roles governments and markets should play in bringing about climate stabilization (Storm 2009). Hence, what I propose to do is to expand Heal and Millner’s (2014) concept of normative economic uncertainty by introducing two economic issues on which reasonable people can reasonably disagree. These disagreements have their origin in different prior beliefs and valuations, which, as Myrdal (1969) argued in his *Objectivity in Social Research*, unavoidably structure social science research. Objectivity for Myrdal means knowledge derived from openly stated value and fact premises—which is not common practice in economics. The first issue (or axis of debate) concerns fundamentally different value judgements on the appropriate roles of “markets” and “state” in bringing about a structural transformation of the economy, which is an age-old dividing line in economic thinking (see Foley 2008). The second issue (axis of debate) concerns different views on the conflict between the “efficiency” of climate action and its implications for “equity”, which is the single most debated issue in climate economics (e.g., Howarth and Norgaard 1990; Gowdy et al. 2010; Arrow et al. 2012). Each axis of debate reflects a continuum of value judgements or belief systems. Combining the two axes (as in Fig. 1) leads me to a four-fold classification of (normative) economic approaches to climate stabilization which, jointly, cover all major schools of thought in contemporary economics (see Foley 2008; Harvey 2015) as well as the key debates in greenhouse economics (Lohmann 2011; Grubb 2014; Wagner and Weitzman 2015). I will first consider each of these two axes separately.

## Market Versus State

The first dimension measures “faith in the capacity of the invisible hand to adjust the natural thermostat.” At one extreme we find (most) mainstream climate economists who want to make the market work for the environment and get the prices right. Theirs is a—what Nordhaus (2007, p. 689) calls—“*simple economic insight*”: by allocating a full set of property rights to the atmosphere (similar in nature to patent rights, copyrights etc.), carbon emissions get a price. This will confront energy users—billions of firms and people—with the expensive reality that burning carbon has a significant external cost that ought to be taken into account by being charged full freight for doing it. The belief is that self-interest and market mechanisms will achieve an efficient, least-cost solution to the climate crisis, regardless of how the target of atmospheric CO_2_ stabilization is set, once the so-called social cost of carbon (SCC) gets internalized in costs and market prices.^5^ In Weitzman’s (2007, p. 723) animated prose,

the *breathtakingly simple vision* [is] that steady pressure from […] a high carbon price reflecting social costs […] would do more to unleash the decentralized power of capitalist [..] inventive genius on the problem of researching, developing, and finally investing in economically efficient carbon-avoiding alternative technologies than all of the piece-meal command-and-control standards and patchwork subsidies making the round […] these days.

Pricing carbon is *transformational*, because the steady pressure from a high carbon price reflecting social costs will “unleash robust private sector investment and innovation to achieve deep emission cuts at least cost” (World Bank 2010, p. 190). It is this “breathtakingly simple vision” which dominates policy responses to climate change by the IEA (2015), the IMF (2008), the UNDP (2007/8), the World Bank (2014), the EU and the IPCC (2014).

In principle, there are two ways in which the SCC can be internalized in prices: by imposing carbon taxes or by a scheme of tradable carbon emissions permits. Taxation amounts to fixing the optimal price of carbon emissions and letting the quantity of emissions endogenously adjust to this price. If alternatively a cap is imposed, the quantity of emissions is fixed, letting the market for emissions permits determine the carbon price. In theory, therefore, carbon taxation offers price certainty, whereas carbon trade offers certainty about the quantity of emissions (when one assumes perfect monitoring). In a “first-best” world featuring perfect information, perfectly competitive markets and no uncertainty, both schemes are equivalent in terms of effectiveness and efficiency—although the (uncompensated) distributional effects of tax and emissions trading policies would be very different (Weitzman 1974; Stiglitz 2008). However, the equivalence breaks down in practice, because there is uncertainty about future climate damages and about the economic costs of reducing emissions. The optimal policy to choose *ex ante* is then the instrument that is expected to exhibit the smaller degree of deadweight welfare loss *ex post* (Weitzman 1974). If great weight is placed upon the small probability of catastrophic outcomes, then for relatively small increases in carbon emissions, there will result large, even catastrophic, increases in (expected risk-weighted) damage costs. It is in this case preferable to fix the quantity of emissions, since only that way we can be certain of avoiding the risk of dangerous climate change. This suggests the superiority of carbon trading, as under carbon taxes, the exact quantity of emission reductions is endogenous and hence uncertain (Pizer 2002; Stiglitz 2008). The case for carbon trade in a “first-best” world thus rests on Weitzman’s (2009, 2013) “fat tails” argument. In addition, there is a widely held view that, in our “second-best world”, carbon trading is the superior system, because trading is politically more acceptable to corporations and consumers than taxation (leave alone direct regulation), and ultimately less costly (Stern 2009; Helm 2010). Indeed, strong opposition from industry blocked attempts to control emissions by direct regulation and taxation in the EU in the 1990s (Christiansen and Wettestad 2003).

Market sceptics beg to disagree and argue that the magic of the market cannot be made to work. For a start, setting the right (optimal) corrective price for carbon is impossible, because we do not have well-defined probability distributions concerning (all) future states of nature (Knight 1921). Uncertainty means we can only evaluate the efficiency of a particular carbon price *ex post*—after the results are in—and, for all practical purposes, the SCC and therefore the *optimal* corrective price of carbon cannot therefore be known *ex ante* (Lipsey 2007). Hence, to argue for market-based instruments on *ex*-*ante* efficiency-grounds is misleading. But even if we reduce uncertainty to merely “model uncertainty”, disagreement about the social discount rate, the climate damage functions and the structure of the climate problem in general leads to a wide variety of estimates of the SCC. To illustrate: relying on 75 studies (and 588 estimates), Tol (2013) obtains a *mean* estimate of the SCC of $53 per ton of CO_2_ (but with a large standard deviation of $88 per ton of CO_2_) and a *median* estimate of $37 per ton of CO_2_. The estimates are particularly sensitive to the social discount rate—with mean estimates increasing up to $80 per ton of CO_2_ for a very low discount rate (see also Ackerman and Finlayson 2006; Johnson and Hope 2012). However, Tol himself, after narrowing down the sample to just 145 estimates (all based on his subjectively preferred high discount rate), concludes that the mean/median SCC is just $6–7 per ton of CO_2_.^6^ Again, we don’t know—although it appears as if Tol’s eagerness to avoid a Type-I error (detecting an impact that is not present) is leading him to understate the much larger risk of running into a Type-II error (a failure to detect the real impact of global warming).

However, even if one could put the right price on carbon emissions, it is difficult to believe that this will bring about the radical—non-marginal—technological progress and social change needed to wean us off carbon. “Economists like to set corrective prices and then be done with it, leaving the rest of household and business decisions to the magic of the market,” writes Sachs (2008), but “this hands-off approach will not work in the case of a major overhaul of energy technology.” Climate stabilization requires a fundamental “disruption” of hydrocarbon energy, production and transportation infrastructures, a massive upsetting of vested interests in fossil-fuel energy and industry, and large-scale mobilization of investment—the International Energy Agency thinks it will cost $44 trillion between now and 2030 (IEA 2015); what is needed is roughly $3 trillion annually, which is ten times more than what the world is spending on climate mitigation now (Table 1). If the carbon price is to incentivize firms to bring about this kind of disruptive change, then it has to be a clear, stable and reliable signal and high enough^7^ to help private firms recover their investment costs plus a substantial premium for market risks associated with deploying uncertain, unproven technologies (Markandya 2009; Newell 2010; Borenstein 2012). Real-life carbon markets are unlikely to produce such a clear stable signal, but in contrast will exhibit disproportionate price volatility as a result of co-ordination failures and information asymmetries (Stiglitz 2008), wrong beliefs about future prices^8^ or short-termism (Georgescu-Roegen 1975; Howarth and Norgaard 1990). Market sceptics therefore tend to reject the notion of “efficiency” in favour of “effectiveness” as the more appropriate criterion for climate policy choice (on grounds of the “no-regrets” motive).

**Table 1:** The relative cost of climate change mitigation

	Billions U.S.$	% of global GDP	Ratio to global investment in climate change
Global investment in climate change 2013	331	0.4	1:1
*Compared with*			
Global fossil-fuel energy subsidies 2015	5302	6.5	16:1
Global military expenditure in 2014	1767	2.3	5:1
Public expenditure on renewable energy in the OECD countries	6	0.008	1:55
Global public investment in climate change 2013	137	0.2	0.4:1
Implicit public subsidy for the 10 world’s biggest banks in 2012	300	0.4	1:1
Global cost associated with obesity	2283	2.8	7:1

*Sources*: data on global and public investment in climate change come from Climate Policy Initiative (2014); on fossil fuel energy subsidy are from Coady et al. (2015); on global military expenditure are from Stockholm International Peace Research Institute *Military Expenditure Database* (http://www.sipri.org/research/armaments/milex/milex_database); on global GDP from the IMF *World Economic Outlook Database*; on public expenditure on renewable energy come from Global Apollo Programme (2015); data on bank subsidies are from Haldane (2012); and data on the global burden due to obesity are from McKinsey (2014). The implicit subsidy to big global banks arises because these system-banks enjoy a government guarantee, because they are “too-big-to-fail”, which improves their credit rating and lowers their borrowing costs

What must be understood is that the radical innovation needed to stop global warming is beyond the capacities of small and even large firms,^9^ because it is very costly, takes at least 20–25 years to fully develop, is an uncertain—non-probabilistic—process with “odds” of success or failure that cannot be objectively calculated in advance, and always features potential gains to society that cannot be appropriated by the innovator.^10^ What is needed, writes the Global Apollo Programme (2015, p. 12), is “the application of basic science to produce fundamental disruptive technical change of the kind we have seen in telecommunications and IT. Those revolutions all began with publicly supported Research, Development and Demonstration.” “No amount of price signals would have created the internet,” write Mazzucato and Perez (2014, p. 14), “just as today a carbon tax or an emissions market would be crucial but not sufficient to get clean tech going.” What is needed is the directional thrust of the state through publicly funded R&D and “mission-oriented” public strategies—as happened with computers, semiconductors, the internet, genetic sequencing, satellite communications, and nuclear power (Block and Keller 2011; Mazzucato 2013; Stiglitz and Lin 2013). States should be entrepreneurial, “market-creating” and “risk-taking”, argue Mazzucato and Perez (2014, p. 14), providing the conditions “that will significantly reduce the risks and increase the potential profitability of what is now technologically feasible but highly uncertain”. These conditions include: carbon taxation to provide the finance for public investments in renewable energy and subsidies for clean technologies, regulatory programmes, intellectual property rights reform and aggressive industrial policy.

Current global investment spending on slowing climate change is just $331 billion (about 0.4 % of global GDP), which is barely a fifth of global military expenditure or one-sixteenth of global subsidies for fossil-fuel energy (Table 1). This has to change in this view (Sachs 2008). Essential is the provision of patient, long-term, committed capital to fund the investments in RD&D—as envisaged by the Global Apollo Programma (2015) which asks governments to pledge to spend at least an annual average of 2 % of GDP as public expenditure on RD&D for renewable energy technologies during 2016–2025. This would amount to $1.6 trillion in 2015, which is 266 times more than the $6 billion the world is now spending on public renewable RD&D. But it may nevertheless be achievable, it is argued, if we compare it to the $5.3 trillion the worlds spends each year on (implicit) subsidies to the fossil-fuel energy sector, the $1.8 trillion of global military expenditure, or the $2.3 trillion cost of obesity-related damages. What would be needed is a *reallocation* of expenditure—not necessarily an increase in spending (and taxation).

## The Second Axis

The vertical axis expresses differences in views on whether it is possible to draw a sharp line between questions concerning the efficiency of climate action and questions concerning its potential distributional or equity implications. At the top of the axis, we have the Coasean perspective (Coase 1960), which holds that issues of efficiency and equity can and must be separated. Efficiency (or maximum utility, attainable when using a given set of endowments of labour, capital and natural resources) is, in this view, the province of economics (and can only be assured by competitive markets, as per the first theorem of welfare economics), while equity (or distributive justice) is argued to fall in the domain of ethics and political discourse (Howarth and Norgaard 1990).^11^ Accordingly, the Coasean view holds that all that matters for the *efficient* internalisation of the external costs of global warming in relative prices is that atmospheric property rights are unequivocally defined and clearly assigned, not whether the distribution is just or unjust. For achieving a predetermined global CO_2_ stabilization target against the least possible costs, that is for achieving an *efficient* outcome, it does not matter how emission rights have been assigned; as Dasgupta and Heal (1979, p. 257), for instance, note: a resource allocation “can be [intertemporally] efficient and yet be perfectly “ghastly” if resource depletion denies future generations the raw materials required to sustain a productive economy.” If the carbon market outcome turns out to be politically or socially unacceptable in distributional terms, corrective action can be undertaken *ex post* (by means of global redistributive lump-sum taxation) or *ex ante* (by a prior re-allocation of carbon emission rights), without interfering with efficient carbon market processes. The latter option is in line with a radical interpretation of the second theorem of neoclassical welfare economics, which states that any desired allocation of utility among the members of a given society can be achieved through the operations of competitive markets *provided* the initial endowments of capital, land and labour are distributed fairly. Importantly, for engaged scholars with progressive orientations, Coase’s proposition makes it possible to argue for an egalitarian allocation of emission rights radically in favour of poor populations in the least developed economies, while maintaining the proverbial efficiency of the market.

At the other extreme we find those who argue instead that efficiency and distribution cannot be separated in a market economy—not even in neoclassical theory itself. The point is perhaps most easily made with reference to “intergenerational distribution” of the costs and benefits of climate change across current and future generations. The hot potato here is the social discount rate *δ*, the rate at which future (net) increments to consumption should be discounted (converting them into present values). A high or even a moderate *δ* means that the present value of far-off future climate damage will be low. For instance, suppose we face unthinkably large climate damages of $10 *trillion* ($10 × 10^12^), expressed in constant 2015 prices, occurring 100 years from now, which will entail inconceivable suffering. This will have a present value of just $320 *billion* at a 3.5 % discount rate, which is less than half the U.S. military budget of 2015. Thus, unless *δ* is very low, the benefits of climate change mitigation in future centuries are almost negligible in present value terms, and any significant short-term expenditures are “too expensive” relative to the (present value) benefits of prevented climate damage (Ackerman and Finlayson 2006; Gerlagh and Liski 2013). We are better off, in this view, just putting the money in a bank and let compound interest rates make us and future generations wealthier, and hence better prepared to deal with far-off climate damages. While there is an extensive “prescriptive” and “descriptive” literature on social discount rates (e.g. Fleurbaey and Zuber 2012) and some consensus on hyperbolic discounting has emerged, choosing the right level of *δ* (i.e. choosing the underlying pure rate of time preference and the elasticity of the marginal utility of consumption) is ultimately a fundamental individual value judgement—and how to combine these judgements into a socially representative preference regarding tradeoffs between the welfare of present and future generations is an inexorable problem of social choice (Spash 2002; Gowdy et al. 2010; Heal and Millner 2014), which is just ignored by the neoclassical model. To further complicate the issue, fairness and decency suggest that a euro of climate damages in a poor region reduces human well-being more than a euro of damages in a rich region—hence, if we decide on moral grounds that the discount rate must be lower for today’s rich as compared to today’s poor—reflecting the fact that a given loss in income has a far greater negative impact on a poor person than on a rich person—then what we do (assuming continued per capita income growth) is to trivialize the climate damage suffered by the poor in the future (Weitzman 2009). Helping the poor now, in other words, goes at the cost of helping the poor in the future. There are no economic guidelines for “balancing” these conflicts—it is an inescapably political and ethical matter. Moreover, “equity” and “efficiency” cannot be separated, because, as for instance Howarth and Nogaard (1990) have shown using overlapping-generations models, there is no fixed notion of “efficiently” valuing global warming damage in neoclassical economic theory. This “efficient” or optimal value of the SCC varies with society’s view of the future, expressed in terms of the social discount rate. The SCC is innately *subjective*: if society chooses *δ* to be high, the SCC will be low and there is little urgency for climate change mitigation; if on the other hand, *δ* is set at a very low level, the SCC will be higher and climate policy may turn cost-efficient. Society’s view of what is intra- or inter-generationally just, determines the level of SCC to be internalized by means of carbon trading or carbon taxation. Ethics comes first, efficiency only afterwards.^12^

In the Coasean view, an “efficient” policy outcome is one that makes the “economic pie” as big as possible and thereby leaves the “winners” in a position to financially compensate the “losers” and still be better off than in a situation without the policy. As a matter of principle, the policy outcome is still considered efficient even if no compensation is actually paid. This is no trivial issue in the case of the climate policy ramp in which temperature is allowed to rise by at least 2.25 °C, since warming will supposedly be a *net benefit* to the world economy until about 2.25 °C of warming has occurred (Tol 2013). The big losers in this scenario will undoubtedly be the world’s three billion poor, who have been least responsible for loading the air with heat-trapping gases^13^ but (living in the “wrong” geographical zones) suffer disproportionally from the loss of ecosystems and biodiversity, on which they are much more directly dependent for their livelihoods than the world’s rich (UNDP 2007/8; Stern 2009; Cline 2007; Gowdy et al. 2010). Any compensation to the world’s poor has to come out of the pockets of the rich—“the Earth’s first-class passengers”—who however are, because they are rich, in a position “to wall themselves off from the rest of humanity” in “green and gated oases of permanent affluence on an otherwise stricken planet”, as Davis (2008) observes. What if the rich decide to fend for themselves? Can the world’s poor and the unborn become politically empowered to ensure a more equitable global burden sharing? We cannot take this for granted. “There is here therefore an antinomy of right against right, both equally bearing the seal of the law of exchange”, wrote Marx (1976, p. 344) in *Capital* (Volume I), and what happens in such a case is that “between equal rights, force decides.”

To throw the issue of ex-post redistribution into sharp relief, note first that many of the three billion “losers” will have actually died due to the climate and economic disruptions and cannot possibly be compensated by those who continue to live—to academically talk about “potential compensation” here is a clear case of misplaced abstractness (Kelleher 2012).^14^ Unfortunately, matters are not more straightforward when it comes to potentially compensating the surviving “losers”. Should we follow market logic and compensate the global poor on the basis of the per-capita incomes or wages they have lost, which for most amounts to < $2 per person per day? If we do, it almost automatically follows that “a given amount of health impairing pollution should be done in the country with the lowest cost, which will be the country with the lowest wages,” as stated by Lawrence Summers in 1991 in an exposed World Bank memo.^15^ The memo concluded that “the economic logic behind dumping a load of toxic waste in the lowest wage country is impeccable and we should face up to that.”^16^ Indeed, what is “efficient” in a market system favours the “first-class passengers”, whose (disposable) income per person is about $71 per day in the OECD countries, and discriminates against the “second-class passengers”, whose losses or damages are counted at a rate of a mere $2 per person per day. It makes, in the non-Coasean view, therefore no sense to claim that markets can deliver “efficient” outcomes: the question is “efficient for whom?” Prices are not distributionally neutral, they are not an unbiased reflection of value based on scarcity or internalized external costs, but are primarily “coefficients of power”. Prices “are set essentially by social relations”, (Galbraith 2008, p. 179) writes, and thus reflect the world’s unequal market power and/or political power relationships.

Even if we leave aside the testing issue of political unwillingness and power, there is a further obdurate problem when it comes to compensating the poorest for the *material* climate damage they are suffering: how should one value—in terms of a common money metric—the loss of ecosystem services, of biodiversity, of human health or even human lives, or of cultural and/or religious symbols? The Coasean idea that the poorest can be financially compensated for the climate-related hardships they suffer, presumes, as Foster et al. (2009) argue, that these various damages are *commensurable* (in terms of a common money metric) as well as *substitutable* for something else, and therefore compensable (see also MacDonald and Corson 2012; Martinez Alier 2015). But what if people hold intrinsic and infinitely large (environmental or human) values, which cannot be compromised and cannot be adequately captured by market values, leave alone traded, and thus fetch no price? “How many tonnes of bauxite is a tribe or a species on the edge of extinction worth?” asks Martinez Alier (2009), “and how can you express such values in terms that a Minister of Finance or a Supreme Court judge can understand? Against the economic logic of euros and dollars, the peasant and tribal languages of valuation go unheeded.” As only prices and profits count, economists can be said to know the price of everything, but the value of nothing (Ackerman and Heinzerling 2004). This same economic logic of euros and dollars is used to convert “nature” into “natural capital”, measurable in money terms—a process that Foster et al. (2009, p. 1091) tellingly call the “Midas Effect”, because it leads to an inability to recognize that there are intrinsic values and critical thresholds in nature which we ignore at our own cost. “Midas turned his daughter into gold in his mad search for wealth. Today’s economics threatens to destroy the lives of future generations as well as those of innumerable other species in like fashion.” By commodifying nature, Coasean economics fantasizes about turning the Earth into money in order to overcome the ecological limits to economic expansion—society as independent of nature, or, as anti-environmentalist economist Julian Simon (1982, p. 207) declared when questioned about the finiteness of the world’s natural resources: “You see, in the end copper and oil come out of our minds. That’s really what they are.” In Simon’s view, everything in nature if exhausted can be substituted for with the help of technology. It means that the natural environment presents no planetary limits to growth (Rockström et al. 2009; Steffen et al. 2015; Vira 2015)—nature simply disappears, as do social-economic and normative value conflicts. Only money matters. The bottom line is simple: in the non-Coasean approach, the global carbon price cannot adequately reflect the true *social costs* of carbon emissions, because the market mechanism does only recognize preferences when these are backed up by purchasing power, while neglecting the basic needs and rights of the income-poor, the non-monetized value of the eco-system and intrinsic human values and indefeasible rights. Clearly, not everything that can be counted counts, and not everything that counts can be counted.

## Economic Approaches to Climate Change

Figure 1 presents the dichotomy in views between market enthusiasts and sceptics, on a continuous scale, on its horizontal axis, and the dichotomy in views on the efficiency/equity of climate change mitigation on the vertical one. The two axes give us four discourses on climate stabilization.

### Quadrant One

The dominant discourse of *Quadrant One* holds that the invisible hand can stop climate change in a least-cost manner by internalizing the SCC into costs and prices, preferably by means of a system of carbon emission rights trading—the high carbon price induces rational adjustments in production, investment and consumption decisions, while unleashing investment in zero-carbon (energy) technologies, again in a socially optimal manner. The overriding concern is with “social efficiency” in a first-best world: bringing about the transformation with the smallest opportunity costs to the current generation while fulfilling its moral obligation to future generations (in terms of bequeathing them with higher wealth as a compensation for a warmer world). As long as efficiency is thus ensured, “no resources are left on the table”, and “winners” can potentially compensate “losers” (without themselves being worse off), the issue of distributive justice can be relegated to the (global) political system or to ethicists. There is a shared acceptance of capitalism’s capacity for self-correction and for endogenous, price-incentive-induced, technological problem-solving. Internal debates within *Quadrant One* center rather narrowly on how to set *δ*, how to come to an economic (imputed) value of climate damages (to biodiversity, ecosystems and human health and mortality) in order to estimate the SCC, how to design and monitor carbon markets, how to incentivize green innovation, and how to deal with the various climate-economy risks (including so-called fat tail risks). As far as the state is concerned, government’s role should be restricted to narrow supply-side economics: helping build carbon markets; subsidizing green RD&D; promoting links between universities, research institutions and firms; and protecting private property rights including carbon credits.

Neoclassical climate economists can be found in *Quadrant One*, including the IAM-builders (Manne et al. 1995; Markandya 2009; Johnson and Hope 2012; Anthoff and Tol 2012; Nordhaus 2013), but also Cline (2007), Dietz and Fankhauser 2012), Weitzman (2009, 2013) and Stern (2007, 2009, 2013). Prominent ecological economists of a neoclassical bend, such as Daly (1996) and Costanza et al. (1997) and representatives from the climate justice movement (Sachs and Santarius 2007; Baer et al. 2009; Chakravarty et al. 2009) share exactly the same “carbon trading” discourse, but do reject the climate policy-ramp (arguing for immediate action) with greater emphasis on the distributive justice of the (efficient) market outcomes (as does Stern 2007, 2013). Key items for internal discussion in *Quadrant One* include how to infer *δ* from individual time preferences (as revealed by the market interest rate), what to do with climate risks (but ignoring uncertainty), or how to make “climate damages” commensurable in a money metric, *ad infinitum*. Their default policy recommendation is to wait and see—and their discussions are, in essence, deeply social, political and ethical disputes being waged under the technocratic cover of mathematical models (IAMs). “The social discount rate should reflect explicitly moral, other-regarding judgements about the relative importance of well-being that exists far in the future,” writes Kelleher (2012, p. 47), but this is the stance of the ethical (ecological) fringe^17^ in *Quadrant One,* which is sidelined by the mainstream majority as being either “paternalistic” or logically impossible (within the neoclassical premises). The one factor which might upset the policy-ramp consensus of *Quadrant One* is the recognition that the (fat-tail) probability of truly disastrous climate change is non-zero and rising fast with rising (median) temperatures—the impact of this will overwhelm the impact of the choice of the social discount rate and is likely to have a disproportionate impact on climate policy choices (Weitzman 2013).

### Quadrant Two

*Quadrant Two* features discourses that favour a global carbon tax in combination with regulation, direct intervention and (equitable) redistribution by means of a global welfare state. By internalizing the SCC in market prices, carbon taxation is argued to help correct the distorted (relative) price system, aligning market incentives and the social costs of economic activity. But corrected prices are not enough to induce the resource mobilization required for the zero-carbon revolution, which exceeds the capacities of private capital, or to reduce the risks and the uncertainty associated with the radical transformation, which are too big to carry for banks and entrepreneurs. The revenues from carbon taxation must be used to finance essential large-scale RD&D and demonstration projects.^18^ The deeper insight here is that because technology is a public good and innovation is radically uncertain, markets need to be guided, perhaps even disciplined, by a “mission-oriented state” so as to bring about the transformation towards eco-friendly industry, fuel saving technology, alternative energy, and clean transport. Such government guidance is argued to improve the social efficiency of the transition process in a second-best context of multiple market failures (although this cannot be unambiguously proven). Distributive justice is still treated as being separate from efficiency (as in *Quadrant One*) and to be addressed by ex-post (lump-sum) taxes and transfers—the latter may include Northern economies paying off their carbon debts to the South (which is three times as large as the conventional financial debt that developing countries owe to the developed ones). Economists in *Quadrant Two* call for a Green New Deal—a programme of tax-financed mission-oriented public investment in green innovation and low-carbon energy and transport infrastructures, regulations imposing green standards, and redistribution to share the burden of the transition. In this quadrant we may locate Stiglitz (2008), Barbier (2009), the Green New Deal Group (2013), Grubb (2014), Herman (2015) and the Global Apollo Programme (2015) as well as Galbraith (2008) and Pollin et al. (2014), even though the latter two argue for deeper reforms (which would place them in *Quadrant Three*).

### Quadrant Three

The discourse in *Quadrant Three* is built around the more radical attempt to “save capitalism from the capitalists”. The belief here is, unlike in *Quadrant Two*, that more is needed than a socially-engineered Big Push towards a zero-carbon economy. This belief is founded upon the recognition that there are “limits to growth”, in the shape of “planetary boundaries” to human economic activity (as stressed by Georgescu-Roegen 1975) as well as intrinsic human values and indefeasible human rights, which should not be ignored or compromised. These limits are “social” or “normative” boundaries, as the Earth will not stop turning once they are transgressed—but transgression will certainly mean “the end of the world as we know it” and monetary compensation cannot ever bring it back. Consider asking the people living in the year 2115 whether they would prefer inheriting $10 trillion in cash (option A) or $10 trillion worth of avoided temperature rise and associated droughts, famines and hurricanes (option B)? Taking the cash means accepting the loss of millions of hectares of rainforest, most of the world’s sea ice, and most of the world’s coral reefs, coming to terms with mass migration, heightened conflicts and increased human mortality and morbidity, and facing an unknown increase in the risk that this degraded biosphere cannot sustain us any longer. Which option would they choose and which option should we anticipate for them? *Quadrant Three* goes for option B,^19^ accepting as a fact that there are ultimately “normative” boundaries on economic growth, and even entertaining the idea that “de-growth” is necessary (Martinez Alier 2009, 2015; Spash 2015). Deep reforms of our economic and political systems are needed to force capitalism back within sustainable boundaries, curtailing and replacing markets (Polanyi 1944; Lohmann 2009), while capitalism’s institutions governing corporations, employment, income formation, technology and knowledge processes, and trade and finance have to be fundamentally reformed to share income, employment, knowledge, and technology in a fair and equitable manner. Key contributions to this discourse include Speth (2008), Victor (2008), Jackson (2009), Lohmann (2009, 2011), Schor (2010), Arsel and Büscher (2012), Kallis et al. (2012), Klein (2014), Vira (2015) and Pope Francis (2015)—which resonate with arguments made in contemporary environmental ethics and in deep ecology in particular (Brennan and Lo 2015).

### Quadrant Four

Unlike the evolutionary approaches in *Quadrant Three* (which hold that capitalism can be gradually reformed, and even transformed, into a zero-carbon system), the revolutionary discourses in *Quadrant Four*, finally, are constituted around the idea of an un-resolvable conflict between capitalism’s inborn drive for growth and ecological sustainability (Foster et al. 2009). O’Connor (1998) has called this “the second contradiction of capital”: ecologically over-exploitative capitalist growth must at some point collapse, because it undermines the Earth’s capacity to reproduce the ecological conditions for such growth. This conflict (inherent in capitalism) can only lead to radical and abrupt change or revolution: the collapse either of the capitalist system (*der große Kladderadatsch*) or of our climate. In the first scenario which has its roots in the climate justice movement, Green socialism (Altvater 2007; Löwy 2007), Marxist thinking (O’Connor 1998) and feminist politics (Mellor 1997; Warren 2015), ecology and anti-globalization movements, there is hope for a better, sustainable and socialist system, in which private property is abolished, natural resources are collectively owned and co-operatively managed, the use of natural resources is subject to decentralized democratic decision-making and people willingly accept a low-consumption, low-growth, high equity model that results in improved welfare, a better quality of life, and greater democratic control of production and (renewable) resources. Key texts on this ecological revolution are Kovel (2007), Magdoff and Foster (2011), Weston (2014) and Foster (2009). In the second scenario, in which O’Connor’s (1998) second contradiction of capitalism is not superseded, there is no redemption and humanity will be going further down the road to Mike Davis’s “first-class, second-class passengers” Earth (see also Weston 2014).

## No Po-Mo

Figure 1 may give the impression that “there are no truths, only interpretations” when it comes to climate change economics. This would be wrong however. For one, the natural reality of accelerating climate change is going to make itself felt soon: on the current alarming trend, the concentration of carbon dioxide in the atmosphere will exceed the critical level of 450 ppm for a 2 °C rise in global average temperature already by 2035, and by the end of the century we will reach 700 ppm (as per IEA’s “New Policies Scenario”). To put this in perspective: ice-core data show that carbon dioxide was never outside a range between 180 and 300 ppm during the last 800,000 years, with instances above 280 ppm exceedingly rare (Weitzman 2009). Carbon concentration is now already far outside its natural range and increasing at a stupendously rapid rate. For lack of analogue, we cannot know what will happen next. If we follow the central IEA forecast, GHG concentrations around 700 ppm will raise median world temperature by 3.4 °C, with already massive consequences including the melting of permafrost areas and substantial rises in sea-level. There is a 50 % probability of higher temperatures—and an 11 % risk of unprecedented warming in excess of 6 °C (which is close to the risk of losing when playing Russian roulette). Such risk of a catastrophic outcome should already be enough to motivate investment to avert climate change even in the face of uncertainty—just as people buy health insurance without knowing if it will pay off (see Ackerman and Finlayson 2006; Weitzman 2009, 2013; Pindyck 2013; Stern 2013; Heal and Millner 2014; Rosen and Günther 2015). There is no escape from a *precautionary* approach to climate policy to avoid the upper tail risks of extreme warming.

A second lesson from Fig. 1 is that the “breathtakingly simple vision” that supposedly “transformational” carbon pricing can do this job is untenable (Lohmann 2009, 2011). Theoretically, there is no “objectively known and correct” social discount rate, which is universally valid (for rich and poor and for current and future generations), and the SCC is a notion based on strong normative and ethically dubious assumptions (see also Hansson 2007). Besides, the idea behind social discounting—converting future costs and benefits into present discounted values—is that society has alternative investment opportunities, whose proxy rate of return is the discount rate, representing alternative capital-accumulation opportunities that would create benefits to compensate humanity for the economic losses suffered from climate change. But in case of *ruinous* climate change, it is difficult to imagine what the compensating investments are and opportunity costing makes no sense. We also cannot analytically separate efficiency and equity and we cannot avoid the Midas Effect if we try to make climate damages commensurable in money terms. Practically, carbon markets cannot be made to work, as also Pope Francis (2015, p. 126) recognizes:

171. The strategy of buying and selling “carbon credits” can lead to a new form of speculation which would not help reduce the emission of polluting gases worldwide. This system seems to provide a quick and easy solution under the guise of a certain commitment to the environment, but in no way does it allow for the radical change which present circumstances require. Rather, it may simply become a ploy which permits maintaining the excessive consumption of some countries and sectors.

Besides, as we know from innovation economics, (carbon) price incentives alone will not be enough to bring about the needed radical technological change—given its uncertainty, the associated scale of disruption and the amount of finance needed for it. Mainstream climate economics exhibits much unwarranted optimism about (carbon-price induced) carbon-saving technological progress and innovation, but at the same time, serious discussions of how difficult it is to finance the carbon revolution are conspicuous by their absence in *Quadrant One*, as the financial sector is not modelled in the IAMs.

In *Quadrant Two* it is understood that in our second-best world, overall efficiency can be improved by appropriate state intervention. “The important thing for Government is not to do things which individuals are doing already,” wrote Keynes (1926), “but to do those things which at present are not done at all.” This is the key lesson to learn from this quadrant—price incentives and a few nudges will not engender the structural change to a low-carbon society (Storm 2015). Hence, the concern is with mission-oriented public investment—financed by carbon taxation and using banks to provide the committed capital to enable private green initiatives—and technology-forcing regulations as the means to steer clear of the worst-case scenarios. Avoiding dangerous climate change requires collective action on a large enough (global) scale as well as on all the “climate stabilization wedges” identified by Pacala and Socolow (2004)—which may require an “entrepreneurial state” which is large enough to provide direction and carry the risks. However, such “Green Keynesianism” suffers from the “optimist’s paradox” which justifies continued economic growth, the use of more resources and the generation of more pollution, by pointing out that our descendants will be better off—even though they will face a degraded environment and a lower quality of life. This paradox arises because of unwarranted techno-optimism and because everything is made commensurable in terms of money—the Midas Effect applies here as well. The techno-optimism just presumes that it will be possible to delink carbon emissions from *economic growth* soon enough. Economic growth is important because it makes it easier to come to an agreement on how to “share the burden of saving the planet” (Stiglitz 2008)—as it offers opportunities for money-metric win–win compensation which will be impossible in a de-growth scenario. All this represents a heroic leap of faith that radically understates the economic costs, technological hurdles, and social changes required to cut down carbon emissions. Hence, approaches in *Quadrant Two*, while being necessary, may not be sufficient to steer humanity clear of the upper-tail risks of extreme warming.

*Quadrants One* and *Two* both offer establishment arguments which seek to address climate change without confronting the reality of capitalist growth (Speth 2008; Weston 2014), and because both will likely fail to bring about the transformation, the material realities of business-as-usual (BAU) capitalist growth, carbon emissions and climate change are bound to end up in the worst-case scenario of runaway warming—as predicted in *Quadrant Four*. This is the story of climate disaster foretold—and its pain will be borne by the poor and the unborn, the “two constituencies with little or no political voice.” It makes little sense to speculate what will happen (or needs to be done) after we have let the climate system go out of control—it will be bad and it is the exact outcome any reasonable person would want to avoid. The very desperation of many of *Quadrant Four*’s arguments highlights the need for more radical measures to stop global warming from becoming dangerous, including calls for “stopping with coal and oil”, “imposing carbon neutrality” and fast geo-engineering the climate which is overall fall-back option (Wagner and Weitzman 2015; but see Buck 2012). On a closer look, however, these radical proposals present the issue of, what Foster et al. (2009) call, revolutionary or transformative change of human societies—which would require not simply (radical) technological progress and more interventionist governments, but far deeper changes in social relations, politics and culture to make the switch to “de-growth”. This brings me to *Quadrant Three* where in my opinion the only lasting solution to the escalating climate crisis lies. “It’s not enough to invent new machines, new regulations, new institutions. We must develop a new understanding of the true purpose of our existence on this Earth,” wrote Vaclav Havel.^20^ This means that we have to discard the idea that it is narrow self-interested behaviour which, guided by the “invisible hand”, will lead to the best possible outcome—it won’t and what we need instead is an enlightened other-regarding “solidarity”. This is indeed utopian—but as Tinbergen (1994, p. 88) wrote: “Some of these proposals are no doubt, far-fetched and beyond the horizon of today’s political possibilities. But the idealists of today often turn out to be the realists of tomorrow.”

## Conclusions

The “pessimism of the intellect” sees much reason for despair—if only because the (carbon pricing) policy recommendations, given by mainstream climate economics (of Quadrants One and Two) which dominate climate policy discussions today, will not be sufficient to avoid the upper-tail risks of extreme warming in future. What needs to be recognized, however, is that the mainstream (policy) consensus is founded on key value judgements, any one of which should be deliberated, debated and scrutinized. Is the market mechanism capable of bringing about the technological transformation toward a zero-carbon economy all by itself and fast enough? If this is not the case, how much and what kinds of government interventions are needed? Can we treat the “efficiency” of the transition to a zero-carbon economy as an issue that is separate from the distributional side of the same process? If this is not the case, how should we balance the trade-off between “efficiency” on the one hand and intra- and inter-generational “equity” on the other? Can we make the manifold climate damages commensurable in a common metric or not? If not, how do we handle intrinsic values and critical (ecological and/or social) thresholds? These are all issues that need to be discussed—and on closer look, they are all inescapably normative and unresolvable by means of more or improved empirical knowledge. What is needed instead are explicit normative frameworks and novel deliberative decision-making frameworks (on these issues) that are appropriate to dealing with fundamentally uncertain climate change featuring “low-probability, catastrophic impact” outcomes (Heal and Millner 2014).

To open up such deliberative space, more will be needed than changing our *interpretation* of the world which in any case will take time. We may need to begin *changing* the BAU scenario already now. We could, for instance, follow Lord Stern’s suggestion (Guardian 2015a, b) to divest from fossil fuel companies and invest in companies that are taking responsible action on climate change. Or one could start more climate liability law suits against governments, as was successfully done by *Urgenda*—a Dutch citizens’ group which accused the Dutch government of negligence for “knowingly contributing” to a breach of the 2 °C maximum target for global warming. Perhaps only such *praxis* will force mainstream climate economics to face up soon enough to the reality of non-negligible upper-tail risks of extreme warming.

## Footnotes

1 In Tol’s (2013) view the “efficient” scenario is the one in which atmospheric concentration of greenhouse gases increases to more than 625 ppm CO_2_-equivalent by 2100; lower targets supposedly have ruinously high costs. Such carbon concentrations in excess carry a risk of more than 6 % of warming in excess of 6 °C (Wagner and Weitzman 2015).

2 See Ackerman and Finlayson (2006), Mastrandrea (2010), Weitzman (2009, 2013), Dietz and Stern (2013), Scrieciu et al. (2013), Pindyck (2013), Revesz et al. (2014), Weyant (2014), Moore and Diaz (2015), and Rosen and Günther (2015).

3 This is not a deterministic or “natural” process but steered or blocked by deliberate policy interventions and hence not politically neutral. Warming may as well slow down technological progress (Moore and Diaz 2015).

4 An example is the technology for capturing and storing CO_2_, which if not deployed soon, will double the cost of stabilizing GHG levels, see IPCC (2014).

5 The SCC is a comprehensive estimate of the economic damages associated with a small increase in CO_2_ emissions, conventionally one metric ton, in a given year. This euro or dollar figure, derived from IAMs, also represents the present (discounted) value of damages *avoided* for a small emission reduction (i.e. the benefit of a reduction).

6 See Ackerman (2014) for a critique of Tol’s method.

7 Borenstein (2012) indicates that the SCC needs to be higher than $86 per ton of CO_2_ to make residential solar PV electricity cost competitive. Based on calculations with the POLES model, Markandya (2009) argues for an even higher carbon cost of $115 per ton of CO_2_ to incentivize private investment.

8 For example, to the cost of their shareholders and taxpayers, U.S. automobile manufacturers misjudged future oil and carbon prices, producing fuel-inefficient cars now no longer in demand and missing the boat on energy-saving technological progress.

9 Banks, financial institutions and venture capitalists are risk-averse, not interested in in-appropriable external effects and focused on the short term—venture capitalist generally exit within three years, usually via an initial public offering (Lazonick and Tulum 2011)—and hence are only willing to fund incremental innovations that ride on existing proven technologies, but not the unproven uncertain radically new ones.

10 The energy sector is one of the least research-intensive of all sectors and the ratio of R&D to sales is under 2 % compared with over 5 % in consumer electronics and 15 % in pharmaceuticals (Global Apollo Programme 2015, p. 13).

11 This holds true only in neoclassical theory of a “first-best” perfectly competitive economy in which all resources are fully employed. In Keynesian theory, in which economic activity is limited by effective demand, “equity” and “efficiency” cannot be separated, because a redistribution of income from high-income groups (who are saving a relatively high proportion of their incomes) to lower-income groups (who are saving less) will raise aggregate demand, boost economic activity and reduce unemployment—in sum: redistribution raises macro-economic efficiency. This way, as Skidelsky (2003, p. 365) writes, “what [Keynes] did was to transfer the problem of social justice from the micro-economy to the macro-economy.”

12 See Brennan (2007) for an analysis of the ethical choices involved in discounting.

13 Evidence by Michael and Vakulabharanam (2015) shows the rich are the major polluters in India. Zwickl et al. (2014) find for the U.S. that low-income minorities have the highest exposure to pollution.

14 See *The* 2015 *Lancet Commission on Health and Climate Change* for an analysis of the global health impacts of a warming world, warning that inaction may set back 50 years of progress in global health.

15 For details, see Ackerman and Heinzerling (2004). Summers claims that he signed the memo but did not write it, that its contents were taken out of context, and that the whole thing was meant as “sarcasm” anyway.

16 This is exactly the logic applied to—what economists euphemistically call—the value of a statistical life (VSL): an equivalence factor based on a person’s wage (as a proxy of one’s contribution to GDP), widely used in climate-policy cost-benefit analyses to transmute climate-change induced increases in human mortality in money terms. See Ackerman and Heinzerling (2004).

17 This ethical fringe (including many from the climate justice movement) fits in *Quadrant One*, because it does assume that carbon pricing can make capitalism self-correct in a socially optimal manner.

18 In addition, public funding for climate mitigation can come from within current budgets by cutting spending on unproductive items. For example, for every dollar allocated for stabilizing the climate, the US government (in 2013) spends $24 on achieving security by military force and for every dollar allocated to developing new technologies to stabilize the climate, it budgeted $7 to develop new weapons systems (Pemberton and Powell 2014).

19 It is true that opting for B could also be based on purely instrumental reasons, but approaches in *Quadrant Three* deplore the loss of the world ‘as we know it” in its own right, independent of the (instrumental) usefulness of what gets lost.

20 Cited by Speth (2008, p. 200).
